# Measurement of Spatial Pulse Wave Velocity by Using a Clip-Type Pulsimeter Equipped with a Hall Sensor and Photoplethysmography

**DOI:** 10.3390/s130404714

**Published:** 2013-04-09

**Authors:** Dong-Hyun Nam, Woo-Beom Lee, You-Sik Hong, Sang-Suk Lee

**Affiliations:** 1 Department of Biofunctional Medicine and Diagnosis, Oriental Medical College, Sangji University, Wonju 220-702, Korea; E-Mail: omdnam@sangji.ac.kr; 2 Department of Computer Science Engineering, Sangji University, Wonju 220-702, Korea; E-Mails: beomlee@sangji.ac.kr (W.-B.L.); yshong@sangji.ac.kr (Y.-S.H.); 3 Department of Oriental Biomedical Engineering, Sangji University, Wonju 220-702, Korea

**Keywords:** clip-type pulsimeter, permanent magnet, Hall sensor, radial artery pulse wave, photoplethysmography (PPG), simultaneous measurement, pulse wave velocity (PWV), spatial pulse wave velocity (SPWV)

## Abstract

A prototype of a clip-type pulsimeter equipped with a magnetic field-sensing semiconductor Hall sensor was developed. It has a permanent magnet attached in the “Chwan” position to the center of a radial artery. The clip-type pulsimeter is composed of a hardware system measuring voltage signals. To measure spatial pulse wave velocity (SPWV), the signal from the radial artery pulsimeter and that from the photoplethysmography (PPG) were simultaneously compared. The pulse wave data from a clinical test of 39 clinical participants (male:female = 25:14) with a mean age of 24.36 (±2.35) years was analyzed. The mean SPWV, which was simultaneously measured from the radial artery pulsimeter and PPG, was 0.8 m/s. We suggest the SPWV results were higher for men than women, because of the better vascularity of terminal tissue in men. The findings of this research may be useful for developing a biomedical signal storage device for a U-health-care system.

## 1. Introduction

The new medical paradigm of the ubiquitous (U) healthcare era has arrived. As a result, conventional medical service providers are expected to provide real-time information on confined diagnosis and treatment. U-health-care is important, not only as a new growth industry for the next generation, but also as a primary means of reducing medical expenses, especially in those countries where medical expenses have been on a continuous and steep rise, owing to an increasing elderly population [1]. Thus, U-health-care development research of a wearable medical device for bio-signals to measure the pulse wave velocity (PWV) and the spatial pulse wave velocity (SPWV) is required [2,3]. Here the SPWV slower than the PWV is the different bio-signal measuring the compliance or the tension level of blood vessels in the circulatory system.

The bio-signal measuring instruments that are currently available on the market are limited in that their time to locate and measure the signal is delayed. Nevertheless, wearing the sensor module on the wrist or forearm allows home use of the medical device. This allows blood pressure monitoring, PWV, and SPWV through bio-signals, thus providing patients with vital information regarding their physical and cardiovascular condition. However, it is difficult to obtain highly accurate blood velocity and SPWV measurements noninvasively without maintaining constant pressure through pressurization. A medical device with low accuracy is not capable of providing the reproducible measurement results required for diagnosis and treatment.

The development of medical devices for bio-signal monitoring of pulse frequency, heart rate, the blood velocity, SPWV, and blood pressure is a prerequisite for the U-health-care industry [4]. Through periodic research on the radial artery, the Hall device was developed to sense magnetic field changes generated by periodic movement. A permanent magnet has been fixed at the radial arterial side. Based on the same operating principles, researchers advanced the existing technology to develop a wrist-wearable clip-type pulsimeter [5]. Therefore, the present research aimed to develop a medical device with the combined capabilities of a photoplethysmography (PPG) and clip-type pulsimeter. The goal was to measure two respective pulse waves simultaneously and to determine the properties and the potential applications of SPWV studies by analyzing the data obtained from the two measurements.

## 2. Acquisition of the Pulse Wave Using the Clip-Type Pulsimeter

**Figure 1.**
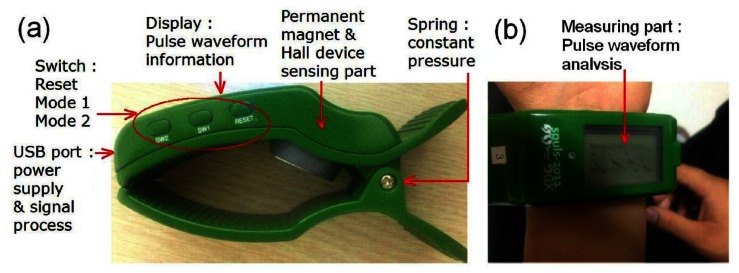
(**a**) The notation and function of several major parts and (**b**) the measurement process of acquisition pulse signals by a real clip-type pulsimeter.

We present here a detailed description of the clip-type pulsimeter prototype used. Figure 1(a) is a picture of the prototype, showing part of the permanent magnet, Hall sensor, partial measurement, Light Emitting Diode (LED) display, Universal Serial Bus (USB) port, and switch. The prototype uses a small cylindrical permanent magnet (diameter, 2 mm; height, 1 mm). This magnet makes contact with the skin and produces a surface magnetic field of 300 Oe and easily fluctuates in response to pulse signals. An elastic latex rubber was used to prevent local pressure on the skin. The permanent magnet is placed at the center of the elastic rubber and fixed with an epoxy adhesive. The latex rubber, placed in the “Chawn” position, is evenly extended from the center of the cylindrical magnet above the radial artery, covering the skin surface of the wrist [3–5]. The distance between the Hall effect device and permanent magnet is approximately 2.5 mm. Magnetic field fluctuation by the high and low vibrations from the artery pulses occurs within 1 mm from the surface of the magnet.

Sample actual measurements of pulse wave signals using our clip-type wrist pulsimeter are shown in Figure 1(b). A pulsatory motion of the radial artery indicates a maximum vertical displacement of approximately 1.13 mm per waveform unit. Therefore, the maximum magnetic field displacement from the skin surface was estimated to be 1.13 mm. Magnetic materials can be easily obtained. In the present study, an ND magnet (Nd-Fe-B-type: Taeyang Magnetech Co., Ltd., Seoul, Korea. Grade: N35, Surface field: Br = 1.2 kOe) with a 3-mm diameter and 1-mm thickness was specifically selected as it was wide enough and easily attached to surfaces where measurements were needed. By determining the deviation in magnetic field intensities between two magnets, we were able to verify that magnetic field intensity measurements vary with magnet type in a distance-dependent manner. The closer the magnet was to the sensor, the greater the deviation [4].

The output obtained by the Hall device presented magnetic field changes to voltage signals, at this time noise signals also changed. The signal was cleaned using an analog filter, and then digitalized by means of an Analog/Digital (A/D) converter. The converter had a built in signal processor that was responsible for accumulating the digitized signals. Sampling the pulse signal to digital signal suggests the feature point of pulse frequency and computing time after differentiating each signal. The magnetic sensor of the Hall device A1395 by Allegro Company (Worcester, UK) has a high sensitivity and has a linear characteristic in strength of the magnetic field [1,3,4]. The *V*_CC_ input voltage has a 10 mV/G sensitivity and let 0.1 V∼3.2 V in the linear characteristic strength of magnetic field when appling *V*_CC_/2 V of polarity. This research used 3.3 V so calculate substitution *V*_CC_ available for sensing upto 155 G. The distance between magnets to sensor has to keep strength of the magnetic field below 155 G. However, the data received by the A/D converter through the analog filter still had noise. So the processor's digital filter was re-designed to remove the noise [5]. The digital filter used a low pass moving average filter to the remove noise. The moving average filter was calculated based on a subject size of 50 adjacent data points. To minimize the noise we increased the filtering area, but that intensified data distortion. Therefore we settled on a more standard filtering area. Each divided section of cycle from the pulse signal has been done by filtering and first derivation.

## 3. Simultaneous Measurement System of Clip-Type Pulsimeter and PPG

The waveform was recorded by using a clip-type pulsimeter that detects the changes in blood vessel waveform and pressure which influences the blood flow from the heart to throughout the entire body. Blood ejected from the left ventricle of the heart is transported through the peripheral blood vessels. The amplitude of the radial artery pulse is caused by a pressure wave. During cardiac diastolic period, partial blood penetration occurs from peripheral tissues in the heart. The minimal change in tissue blood volume after each cardiac cycle can be recorded using various methods, a few of these are electrical, mechanical, and optical methods, among others [6].

**Figure 2.**
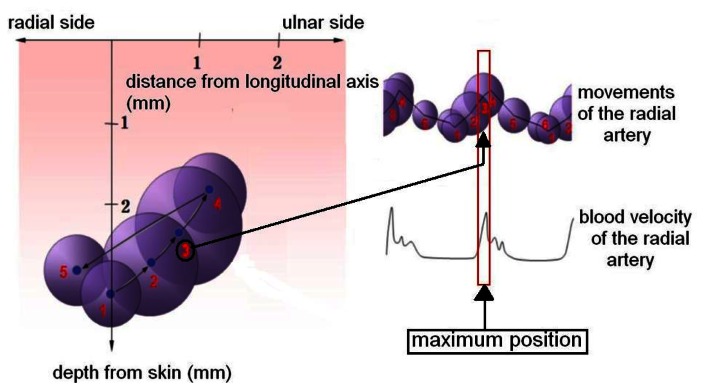
Schematic diagram of the three-dimensional motion of the radial artery, showing the relation between arterial pulse motionand blood flow in the radial artery. Here the maximum point means the largest diameter and blood velocity of the radial artery.

The pulse waveform signal is influenced by the bending angle of the wrist and by the “Chawn” position of the sensor in the radial artery pulsimeter. The permanent magnet, when suitably placed in the “Chawn” position across the radial artery, responds to pulsatory motion by displacement of the magnetic field. As with other existing pressure, infrared rays, and impedance sensors, the Hall sensor can be used in radial artery pulsimeters to convert pulse signals [7]. The change in waveform depth from the skin surface resulting from the change in the blood vessel thickness and its jiggling as blood flows through it indicates the periodic work (*i.e.*, per cardiac cycle) of a blood vessel. Figure 2 illustrates the changes in radial artery movement and blood velocity [6]. Figure 2 also shows that the arterial pulse motion is related to the blood velocity in the radial artery. The blood velocity increases as the pulse amplitude and diameter of radial artery increase.

PPG, which measures the degree of light absorption in a tissue based on the change in peripheral blood flow rate, is used in the optical method of measuring pulse waves [8,9]. If we measure the intensity of the light transmitted to the skin by attaching a luminescent sensor and photo detector to the pulsimeter, the output of the photo detector will indicate that the signals are moving in accordance with every heartbeat. In contrast, other medical devices for relaxation pulse detection indicate an increase in the signals detected with a decrease in blood flow rate. A PPG usually displays a low-frequency band; however, a high-frequency band can occur relative to the origin of the PPG signal, depending on individual pulse frequency [8].

Therefore, the PPG was designed to detect frequency bands ranging from 0.05 Hz to 20 Hz. A direct constant-current source is used to activate the red light of the LED sensor. The light sensor of the photo detector displays two outputs (+, −) with opposite current flow directions by converting the outputs into currents after detecting the incoming light. To convert the currents into voltage, a current-voltage converter circuit is used, connecting the output to a difference amplifier [9].

**Figure 3.**
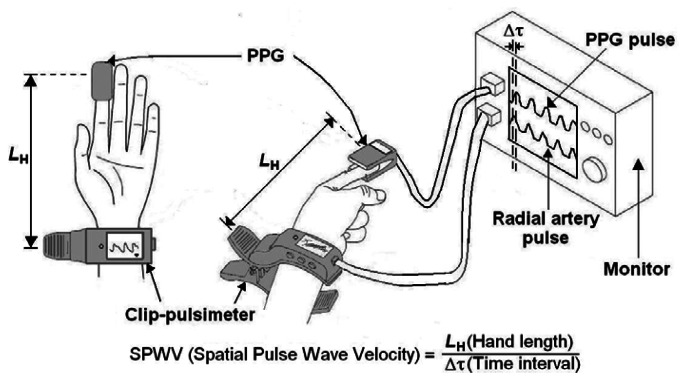
A schematic diagram of the simultaneous SPWV measurement method using a radial artery pulse waveform and PPG pulse waveform. The SPWV defines SPWV = *L*_H_/Δτ. Here, *L*_H_ and Δτ are a hand length from artery wrist to index finger and a time interval of phase difference between two peaks of pulse waveforms, respectively.

A blocking signal was amplified five times at a low frequency of 0.05 Hz after passing through the secondary drudgery-pass filter of the differential motion amplifier. Then it was amplified a hundred times at a minimum frequency of 20 Hz after passing through a secondary low band-pass filter. In our present research, we measured SPWV using radial artery and capacity pulse waves. SPWV was measured based on the PPG and radial artery pulse wave simultaneously obtained from the PPG worn around the index fingertip and the clip-type pulsimeter worn around the wrist. SPWV was calculated by analyzing the time difference between the peak values of the two pulse waveforms (Figure 3). Also, as seen in Figure 3, the equation for spatial pulse wave velocity is represented by SPWV = *L*_H_/Δτ. Here, *L*_H_ and Δτ are a hand length from artery wrist to index finger and a time interval of phase difference between the two peaks of pulse waveforms, respectively.

An outline of the process was recorded using signal processor hardware. The required power supply of the system hardware was produced by using a transformer for medical use. A microcontroller transmits the Personal Computer (PC) data to an RS-232 communication port, which transmits the waveform to the Liquid Crystal Display (LCD) after acquiring the capacity and radial artery pulse waves by serial communication. The system hardware used to measure bio-signals was composed of four primary parts, namely, a pulse wave measurement device, radial artery pulse wave measurement device, and microcontroller and a PC for data storage.

## 4. Measurement and Analysis of SPWV

When the heart contracts, a pressure wave will occur at the aorta and is delivered to the radial artery. Given the distance of 0.8 m from the muscle to the peripheral parts, it will take the pulse wave approximately 0.23 s to travel to the radial artery, so the PWV would be 3.2 m/s [10,11].

**Figure 4.**
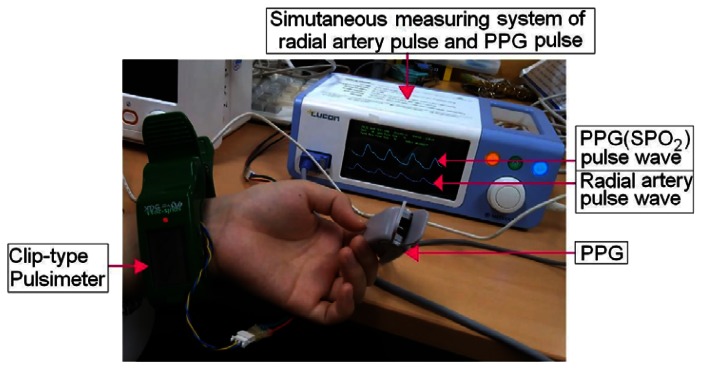
Radial artery pulse and PPG waves obtained from the simultaneous measurements by the clip-type pulsimeter and the PPG meter mounted on the wrist and fingertip, respectively, of the subject's left.

**Figure 5.**
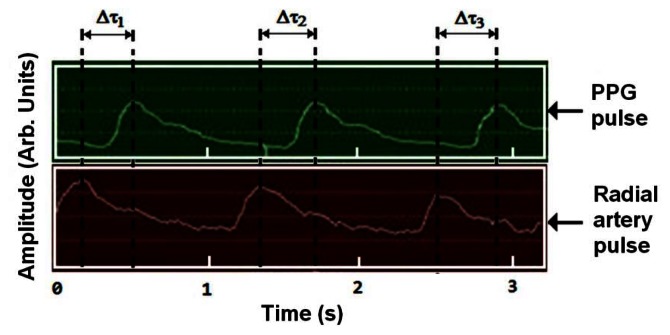
Radial artery pulse graph and PPG signals obtained from the simultaneous measurements by the clip-type pulsimeter and PPG meter mounted on the wrist and fingertip of the left hand. Here, Δτ_i_ is the time interval measured from ith phase difference of the two pulse waveforms.

Using the clip-type pulsimeter equipped with a Hall device, the differences between the two respective waveforms obtained from the PPG, which uses SpO_2_ to indicate the pulse waveform, and the radial artery pulsimeter, which indicates the oxygen saturation level, are simultaneously measured (Figure 4). We were able to measure SPWV by dividing the time difference of the two waveforms by the distance of the wrist to the fingertip. The interrelation of the estimated blood pressure can be determined by performing a statistical analysis of the experimental clinical data [4,5]. In effect, Δτ indicated an approximately three-fold difference in the two peak values (PPG and radial artery pulses in Figure 5).

In our experiment, we measured the distance from sternal angle to wrist, distance from wrist to fingertip, systolic/diastolic blood pressure and pulse rate from 39 clinical participants (male:female = 25:14). The mean age of the participants was 24.36 (±2.35) years old. There was no significant age difference between males and females. The pulse measurements were performed while the subject was in a comfortable sitting position (Figure 4). The clip-type pulsimeter was worn so as to allow as much skin contact as possible for better detection of the pulse signals from the radial artery. The PPG signal was obtained by positioning the PPG sensor on the fingertip of the left index. Hand length (*L*_H_) used to indicate the distance of the left index fingertip and the wrist from the radial artery; and time interval (Δτ_i_), to indicate the time difference between the peak values of the waveforms from the radial artery pulsimeter and the PPG.

**Figure 6.**
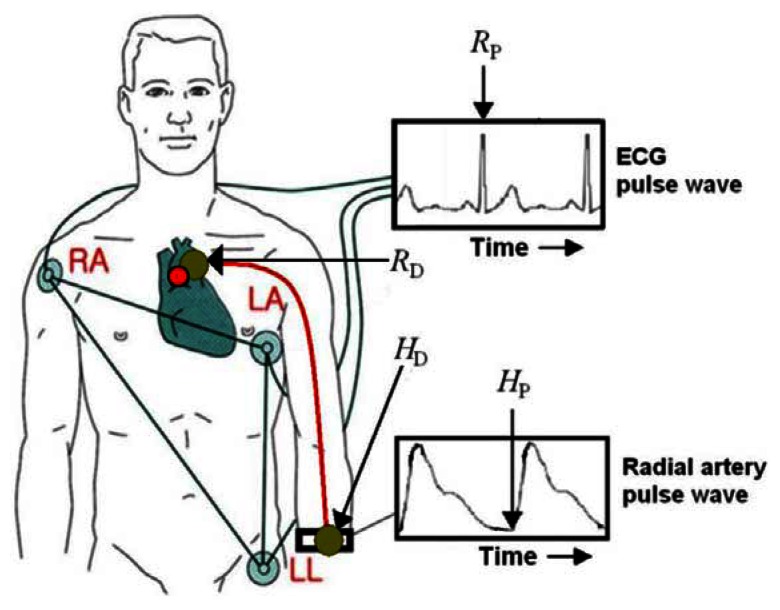
Configuration of electrocardiograph (ECG) and radial artery pulsimeter for the measurement of PWV. Here *R*_P_, *R*_D_, *H*_P_, and *H*_D_ are maximum peaks of the ECG pulse waves, distance of aortic valve position, starting point of radial artery pulse wave, and distance of radial wrist position, respectively. Here, the red filled circle and the red solid line are the exact position of aortic valve and the rough distance from aortic valve to wrist, respectively.

Figure 6 shows configuration of electrocardiograph (ECG) and radial artery pulsimeter for the measurement of PWV. Here *R*_P_, *R*_D_, *H*_P_, and *H*_D_ are maximum peak of ECG pulse wave, distance of aortic valve position, starting point of radial artery pulse wave, and distance of radial wrist position, respectively. The distance between the heart and the wrist of measuring point to get *PWV* = *R_D_* − *H_D_*/*PTT* is *R_D_* − *H_D_*. The exact position of aortic valve and the rough distance from aortic valve to wrist are marked in human body of Figure 6 by the red filled circle and the red solid line, respectively. Because the long axis of aortic valve is nearly located on the sagittal plane, the location of the aortic valve was replaced by the sternal angle which can be easily palpated from the body surface. Here the pulse transfer time (PTT) is the time difference of peak point of ECG wave and start value of the radial artery.

The differences of SPWV and PWV between males and females are shown in Table 1. The significance level was set at *p*-values less than 0.05. The SPWV of males were significantly faster than that of females. And there was nearly significant difference between male and female. In order to investigate the cause of the gender difference, we analyzed the interrelation of SPWV to PWV, distances, blood pressure and pulse rate. The relationships are presented in Table 2.

There was no significant correlation between SPWV and PWV, implying that the clinical characteristics of SPWV are different from those of PWV. In this study PWV displayed, as it is well known, a significant relation to blood pressure and pulse rate [12]. On the other hand, SPWV did not have a significant relation to blood pressure and pulse rate, but rather to arm and hand length. The reason for this result seems to be that the blood pressure inside the arterial vessel will be close to 0 mmHg around the peripheral blood vessels. PWV is mainly determined by collagenization of elastin fibers located in the large artery wall and vascular smooth muscle tone controlled by sympathetic nerve activity [13], therefore an increase in sympathetic nerve activity causes an increase in the pulse rate, blood pressure and PWV, but in case of vascular walls located between wrist and fingertip, especially in the termini of the blood vessels such as capillaries, there are only a few elastin fibers and vascular smooth muscles. Generally blood vessels in men are better developed than in women because men are bigger than women and need more blood supplied to the terminal tissue in body. Consequently we estimated that the reason the men's SPWV was faster than women's SPWV could be that vascularity is better developed in men's terminal tissues.

## 5. Conclusions

In this study, we placed a clip-type pulsimeter, affixed with a permanent magnet on the radial artery protrusion of the wrist to detect through a Hall device the pulse waves that were emitted from the magnetic field generated the work of the radial artery. In other words, a hardware-based voltage detection system was applied by equipping the Hall device on the right upper side after placing a permanent magnet in the center of the radial artery (the “Chawn” area of wrist). We developed a system that used a clip-type pulsimeter and PPG equipment. The SPWV gained by simultaneous measurement, using both the clip-type pulsimeter and PPG, was not in relation to PWV.

Male SPWV was significantly faster than that of females. The difference between that of the males and the females was nearly significant. We believe the reason for the higher SPWV in males is due to the larger vascularity of male terminal tissue when compared to that of females. We suggest these results are the basis for a new bio-signal that can be monitored using a clip-type pulsimeter and PPG and then displayed using a dual screen apparatus for patient to principal clinical parameters. The findings of the present study indicate that SPWV measurements can be useful for obtaining continuous blood pressure and pulse measurement data, using an unpressurized type of PPG for application in a U-health-care bio-monitoring system. However, further analysis of the pulse wave algorithm is necessary to verify the accuracy of our device.

## Figures and Tables

**Table 1.** Differences of SPWV and PWV between males and females.

**Parameters**	**Male (n = 25)**	**Female (n = 14)**	***p*-Value**
SPWV (m/s)	0.84 ± 0.15	0.74 ± 0.14	0.042
PWV (m/s)	7.10 ± 0.79	6.67 ± 0.56	0.088
Distance from sternal angle to wrist (cm)	80.0 ± 1.9	74.4 ± 3.0	<0.001
Distance from wrist to fingertip (cm)	18.1 ± 0.8	16.7 ± 1.3	<0.001
Systolic blood pressure (mmHg)	128.0 ± 11.2	114.0 ± 8.7	<0.001
Diastolic blood pressure (mmHg)	71.0 ± 8.1	66.1 ± 6.3	0.059
Pulse rate (bpm) *	78.5 ± 10.9	76.6 ± 6.4	0.660

Independent samples t-test was performed for difference analysis according to gender.

* Mann-Whitney U test was used for analysis due to equal variance assumption.

**Table 2.** Correlation Coefficients between SPWV and the Other Parameters (n = 39).

**Parameters**	**SPWV**	**PWV**
Distance from sternal angle to wrist	0.455 **	0.253
Distance from wrist to fingertip	0.352 *	0.203
Systolic blood pressure	0.039	0.374 *
Diastolic blood pressure	0.020	0.368 *
Pulse rate	−0.025	0.411 **
PWV	0.106	-

** *p* < 0.01,

* *p* < 0.05.
